# Strengthening value-based infectious disease surveillance in primary health care, Saudi Arabia

**DOI:** 10.11604/pamj.2025.52.85.49422

**Published:** 2025-10-24

**Authors:** Nariman Adeeb AlShakhis

**Affiliations:** 1 Primary Health Care Surveillance Unit, Dammam Health Network, Eastern Health Cluster, Dammam, Saudi Arabia

**Keywords:** Surveillance, value-based health care, infectious diseases

## Abstract

Infectious diseases remain a significant public health challenge in Saudi Arabia and other low-and middle-income countries. While the Kingdom has invested in advanced digital platforms such as Raqueem, HESN, and NPHIES, surveillance practices remain largely compliance-driven, resulting in delays, inefficiencies, and limited engagement in primary health care (PHC). A value-based model can transform surveillance into a resilient, outcome-driven system aligned with Saudi Vision 2030 and International Health Regulations (IHR). This commentary proposes a framework that integrates outcome indicators, digital platforms, and community perspectives, operationalised through a value-based balanced scorecard (VBSC). Key policy options include implementing outcome-based KPIs, strengthening digital integration, enhancing workforce capacity, regular monitoring and evaluation, promoting inter-sectoral coordination, and addressing barriers through policy support. The framework bridges compliance with value generation, ensures cost-effectiveness, and enhances public trust. It provides a replicable model for other low-and middle-income countries seeking to modernise their surveillance systems while improving efficiency, resilience, and accountability.

## Commentary

Infectious disease surveillance is a cornerstone of public health systems, enabling early detection, timely response, and effective containment of outbreaks. However, conventional surveillance approaches are often compliance-driven, emphasising data collection and reporting rather than outcomes and value to patients, populations, and health systems. The global shift toward value-based healthcare highlights the need to reorient surveillance systems toward maximising health outcomes relative to the resources used. In Saudi Arabia, the Health Sector Transformation Program under Vision 2030 emphasises efficiency, patient-centred care, and measurable improvements in population health, principles that align closely with value-based care. Despite significant investments in digital health infrastructure, including Raqueem, HESN, NPHIES, and ANAT, surveillance practices remain fragmented and underutilised. This commentary presents a value-based surveillance system at the PHC level. A value-based surveillance system integrates digital platforms, governance mechanisms, and community engagement, guided by a VBSC. The VBSC enables monitoring across four domains: financial (cost-effectiveness and return on investment), patient/community (satisfaction, trust, vaccination coverage), internal processes (timeliness and completeness of reports), and learning and growth (training and digital literacy).

Policy options include: 1) adopting a value-based infectious disease surveillance framework; 2) strengthening early detection and reporting systems through integrated digital tools; 3) enhancing workforce capacity through targeted training; 4) regular monitoring and evaluation using standardized KPIs; 5) promoting inter-sectoral coordination and stakeholder engagement, and 6) addressing barriers such as fragmented systems and limited resources through targeted policy support [1-8]. Table 1 summarises the proposed policy options for implementing a value-based infectious disease surveillance framework, highlighting their advantages and the potential challenges to adoption. Strengths of this approach include bridging compliance with value generation, aligning with Vision 2030 goals, and providing a scalable model for other LMICs. Challenges include ensuring interoperability between systems, training gaps among PHC staff, and the difficulty of measuring community trust. Future steps involve pilot testing, refining outcome indicators such as detection-to-response time, and conducting economic evaluations to demonstrate cost-effectiveness.

**Table 1:** policy options for value-based surveillance

Policy Option	Advantages	Challenges
Adopt a Value-Based Surveillance Framework	Improves accountability, resource allocation, and benchmarks performance	Requires redesign, staff training, and possible resistance
Strengthen Digital Reporting Integration	Accelerates detection, reduces delays, supports coordination	High initial IT cost, interoperability challenges
Enhance Workforce Capacity	Improves knowledge, adherence, and data quality	Requires ongoing training, possible service disruption
Regular Monitoring and KPIs	Provides accountability, identifies gaps, and supports evidence-based policy	Needs dedicated staff, time-consuming data collection
Promote Inter-sectoral Coordination	Strengthens preparedness, fosters trust, and improves response	Coordination complexity, accountability issues
Address Barriers via Policy Support	Supports adoption, continuous improvement	Needs political commitment, stakeholder buy-in

In conclusion, adopting a value-based surveillance framework within PHC offers Saudi Arabia an opportunity to move beyond compliance-driven reporting toward a system that is efficient, outcome-oriented, and trusted by the public. By integrating PHC into national digital platforms, unifying data flows, and applying a Balanced Scorecard of KPIs, surveillance can become a strategic driver of health system resilience [3,4].
